# Digital connection: effect of online social capital and associated factors on adolescents’ oral health-related quality of life

**DOI:** 10.1590/1807-3107bor-2026.vol40.026

**Published:** 2026-06-01

**Authors:** Eduarda da Silveira BORSTMANN, Thiago Machado ARDENGHI, Mario Vianna VETTORE, Thaís Gioda NORONHA-RAMOS, Bruno EMMANUELLI, Jessica Klöckner KNORST

**Affiliations:** (a) Universidade Federal de Santa Maria – UFSM, School of Dentistry, Department of Stomatology, Santa Maria, RS, Brazil.; (b) Aarhus University, Department of Dentistry and Oral Health, Aarhus, Denmark.

**Keywords:** Social Capital, Adolescent, Quality of Life, Social Support, Online Social Networking

## Abstract

Social capital plays a key role in health outcomes, including oral health-related quality of life (OHRQoL). With increased digital interactions, online social capital has emerged as a potential determinant of adolescent well-being. This study aimed to explore the relationship between online social capital, associated factors, and OHRQoL among adolescents. This cross-sectional study was nested within a cohort study initiated in 2010 with a representative sample of preschool children from Santa Maria, Brazil. Individuals were re-evaluated from 2022 to 2023 (n = 406), when they were between 14 and 18 years of age. OHRQoL was assessed using the Child Perception Questionnaire (CPQ 11-14). Online social capital was measured using two proxy indicators (frequency of social media use and perceived online trust) based on prior literature. Offline social capital and demographic, socioeconomic, psychosocial, and clinical variables were considered confounders. Poisson regression was used to assess associations. Results are reported as rate ratios (RR) and 95% confidence intervals (95% CI). Most adolescents (95.3%) used social media daily, and 62.9% reported having trusted online contacts. Adolescents with low online social trust had CPQ11–14 scores 8% higher (RR = 1.08; 95%CI: 1.01–1.15), indicating poorer OHRQoL. Low offline social capital was also associated with poorer OHRQoL, whereas moderate and high sense of coherence (SOC) were associated with lower impacts on OHRQoL. Social media usage frequency showed no significant association with OHRQoL. Our findings suggest that online social trust, offline social capital, and SOC are positively associated with adolescents’ OHRQoL.

## Introduction

Oral health-related quality of life (OHRQoL) is a multidimensional construct that reflects the impact of oral health on individuals’ daily functioning and well-being.^1-3^ Beyond clinical indicators, OHRQoL is shaped by a range of individual and contextual factors, including social determinants such as social capital.^4-6^ Social capital has been associated with health-related behaviors, access to care, and individuals’ perception of their oral health,^5,7^ emphasizing that oral health encompasses physical and psychosocial aspects.^8,9^ This broader understanding of oral health highlights the importance of considering the social environment and support networks when evaluating and improving OHRQoL, as these elements can significantly influence both preventive care practices and individuals’ subjective experiences of their oral health status.^8,9^

The concept of social capital refers to the resources individuals obtain through their social relationships, networks, and norms of reciprocity and trust.^10-13^ It is commonly divided into two main dimensions: structural social capital, which relates to the extent and frequency of participation in social networks and group membership, and cognitive social capital, which encompasses subjective aspects such as trust, shared norms, and perceived support.^9,14^ Both have been consistently linked to better health outcomes, including in oral health contexts.^5,7^

With the widespread adoption of digital technologies, this concept has expanded into virtual environments. Online social capital emerges as an extension of traditional social capital, referring to the social resources that individual’s access through digital platforms and virtual connections.^15,16^ These online interactions can promote health by enabling access to emotional support, information sharing, and social engagement.^17,18^ Although online and offline social capital share core theoretical components, the formation and maintenance of online ties often differ due to the more fluid, asynchronous, and sometimes anonymous nature of digital interactions.^19,20^

Adolescents, in particular, are immersed in digital environments during a critical phase of development marked by psychosocial changes and evolving social structures.^21^ The COVID-19 pandemic further intensified adolescents’ reliance on online interactions,^22-24^ reinforcing the importance of understanding how digital forms of social capital may influence health perceptions and outcomes. While previous studies have explored the relationship between social capital and OHRQoL,^4-7,25^ most have focused exclusively on offline dimensions.

The complexity of online social relationships and the steady increase in digital use suggest the importance of understanding how social determinants in an online environment possibly influence adolescent population health. Therefore, this study evaluated the relationship between online social capital, associated factors, and adolescents’ OHRQoL. Our conceptual hypothesis was that adolescents with low online social capital are more likely to report poorer OHRQoL.

## Methods

This study adheres to the Strengthening the Reporting of Observational Studies in Epidemiology (STROBE) guidelines.

### Population and sample

This is a cross-sectional study nested within a twelve-year follow-up cohort conducted in Santa Maria, a city in southern Brazil. The estimated population in 2022 was 271,735, including approximately 32,184 school-aged adolescents (10–19 years).^26^

**Figure 1:**
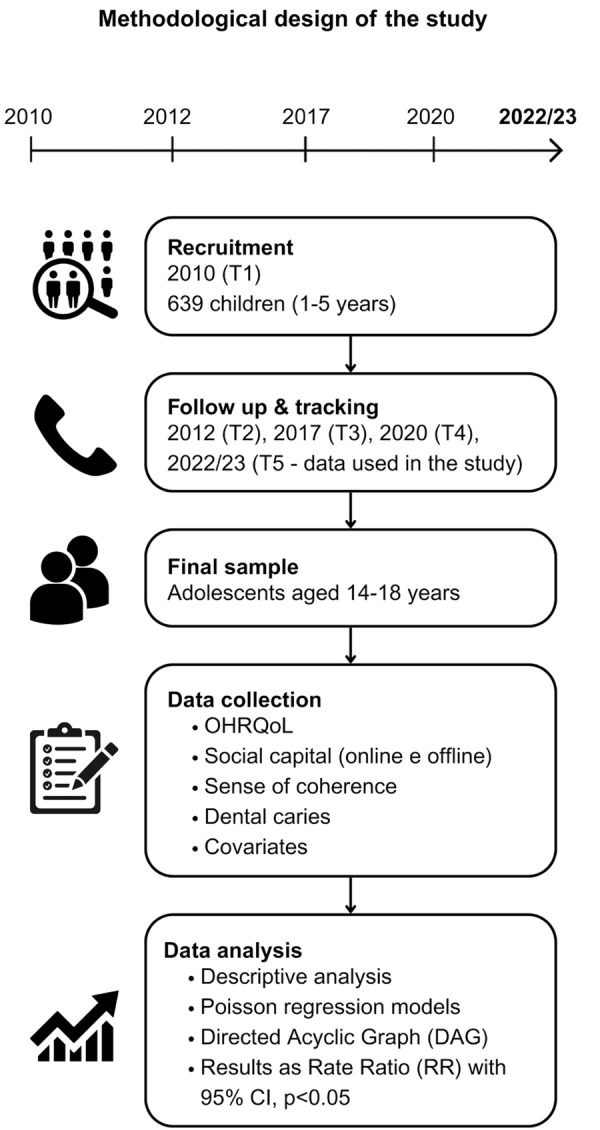
Study flowchart.

Baseline data collection for this cohort took place in 2010 (T1). All primary health care units equipped with dental chairs (n = 15) across different city neighborhoods were included. Children were systematically selected from vaccination queues to participate in the study, and a total of 639 children aged 1–5 years were evaluated. Further methodological details of this stage have been previously published.^27^ Participants were subsequently re-evaluated in 2012, 2017, 2020, and 2022–2023, totaling 12 years of follow-up. All baseline participants (n = 639) were invited to take part in follow-ups. This study analyzed data collected over this 12-year period, during which the adolescents were between 14 and 18 years old (Figure 1).

Multiple search strategies were implemented to minimize participant attrition. Families were contacted via phone calls, searches in the city’s public school enrollment center, and home visits using previously collected addresses. Additionally, social media platforms such as Instagram and Facebook were employed for communication. Examinations and questionnaires were administered at schools or in participants’ homes.

### Collection of data and variables

OHRQoL was assessed using the validated short version of the Child Perception Questionnaire (CPQ 11-14).^28,29^ This questionnaire consists of 16 items grouped into four domains: oral symptoms, functional limitations, emotional well-being, and social well-being. Each item is rated on a Likert scale from 0 to 4: (0) never, (1) once or twice a month, (2) sometimes, (3) often, and (4) every day/almost every day. The total score, obtained by summing all item scores, ranges from 0 to 64, with higher scores indicating a greater impact of oral health conditions on quality of life.

Offline social capital was assessed using questions answered by adolescents: (1) “Have you visited any friend, neighbor, or family member, or have any of them visited you in the past 12 months?” with response options: (0) “yes, at least once a month”, (1) “yes, less than once a month”, (2) “almost never”, and (3) “never”. For analysis, responses “less than once a month”, “almost never”, and “never” were combined into one category. (2) “If something unfortunate happened to you, would someone help you?” with response options: (0) “yes” and (1) “no”. These measures have been widely reported in the literature.^14^

Online social capital was assessed through questions regarding social media use, such as “How often do you use social networks (e.g., Instagram, WhatsApp) to communicate with friends or family?” with response options: (0) “every day or almost every day”, (1) “several times a week”, (2) “rarely”, and (3) “never/do not use social networks”^15,16^. For analysis, responses “every day or almost every day” and “several times a week” were merged. Another question asked, “Do you communicate online (e.g., Instagram, WhatsApp) with someone you trust to help you solve personal problems?” with response options: (0) “yes” and (1) “no”^15,16^. These questions serve as proxy measures of individual-level online social capital and have been utilized in previous studies.^15,16^ Furthermore, they encompass the structural and cognitive dimensions of social capital.^9,14^ Sociodemographic, psychosocial, and dental caries variables were considered potential confounders. Demographic and socioeconomic variables included sex (male or female) and family income (collected in Brazilian reais and categorized into quartiles). Sense of coherence (SOC) was measured using the abbreviated 13-item Sense of Coherence scale (SOC-13), originally developed by Antonovsky and subsequently cross-culturally validated in Brazil to assess individual SOC.^30^ Each item was rated on a 5-point Likert scale (1–5), with a total score ranging from 13 to 65, where higher scores indicate stronger SOC. For analysis, SOC-13 scores were categorized into low, moderate, and high tertiles.

Dental caries were assessed using the International Caries Detection and Assessment System (ICDAS) criteria.^31^ Eight trained and calibrated examiners participated in the clinical data collection. They underwent prior training and calibration, achieving inter- and intra-examiner kappa coefficients ranging from 0.70 to 0.81 and 0.70 to 0.88, respectively. Examinations were conducted individually using gauze, a CPI probe (ballpoint), and a dental mirror. Adolescents were examined at home or school under natural light. For analysis, cavitated caries lesions were classified as present (ICDAS scores of 3, 5, and 6) or absent (scores of 0, 1, 2, and 4).

### Ethical aspects

This study was approved by the Research Ethics Committee (CEP) of the Federal University of Santa Maria (CAAE 63937422.0.0000.5346) and authorized by municipal and regional education authorities. Written informed consent was obtained from guardians, and adolescents provided assent before participation.

### Data analysis

Data analysis was conducted using Stata version 17.0 (Stata Corporation, College Station, USA). Descriptive analysis was performed to summarize sample characteristics. To evaluate sample representativeness after twelve years, participants who completed the study were compared with dropouts using chi-square and t-tests. The study outcome was the total CPQ11-14 score.

**Figure 2:**
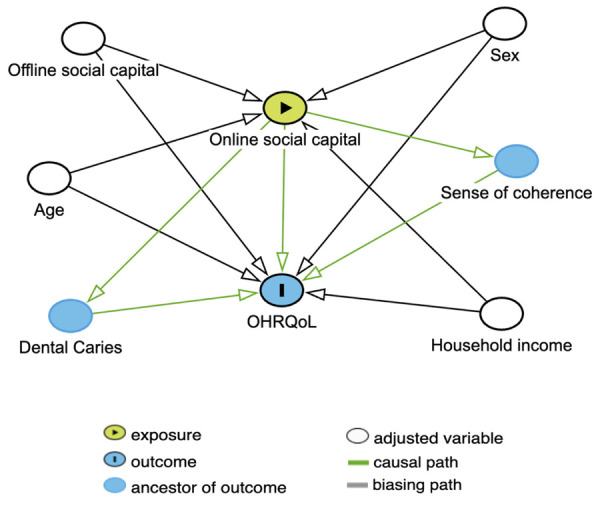
Directed acyclic graph (DAG) with a conceptual representation among the relationship of online social capital, OHRQoL, and possible confounding factors.

A descriptive analysis of sample characteristics according to overall CPQ11-14 scores was performed. Non-adjusted and adjusted Poisson regression models were used to investigate associations between online social capital variables, confounding factors, and CPQ11-14 scores. Potential confounding variables were identified based on previous literature on social capital and OHRQoL, encompassing demographic, socioeconomic, psychosocial, and clinical factors. A directed acyclic graph (DAG) was also constructed to guide the identification of potential confounders and covariates (Figure 2). Variables presenting a p-value ≤ 0.25 in the unadjusted analyses were included in the multivariable model. Results are presented as rate ratio (RR) with 95% confidence intervals (95% CIs). Statistical significance was established at p < 0.05. Model fit was assessed using Pearson’s chi-square statistic, deviance, and the Akaike Information Criterion (AIC), allowing comparisons between crude and adjusted models.

## Results

Among the 639 individuals evaluated at baseline, 406 were reassessed at the 12-year follow-up, resulting in a cohort retention rate of 63.5%. There were no significant differences between those who completed the follow-up and those lost to follow-up, ensuring the representativeness of the original sample.

Table 1 presents the main characteristics of the adolescents. The sample was gender-balanced. Regarding offline social capital, the majority (91.3%) visited or received visits from friends and family at least once a month, and 92.4% reported having social support during difficult times. For online social capital, most participants (95.3%) reported using social media daily or almost daily, and 62.9% had someone they trusted online. The prevalence of cavitated dental caries lesions was 36.0%.

**Table 1:** Demographic, socioeconomic, psychosocial and clinical variables of the sample from Santa Maria, RS (n = 406).

Variables	n*	%
Demographic and socioeconomic variables		
Sex		
Boys	197	48.5
Girls	209	51.5
Age		
14–15	249	62.7
16–18	148	37.3
Household income quartiles in reais (R$)		
Quartile 1 Lowest	102	28.0
Quartile 2	92	25.3
Quartile 3	92	25.3
Quartile 4 Highest	78	21.4
Offline social capital		
Visit to friends/neighbors		
At least once a month	369	91.3
Less than once a month/never	35	8.7
Support during hard times		
Yes	374	92.4
Not	31	7.6
Online social capital		
Frequency of use of social media		
Every day or almost every day	386	95.3
Rarely	11	2.7
Never	8	2.0
Online social trust		
Yes	254	62.9
Not	150	37.1
Psychosocial variable		
Sense of coherence		
Low	101	25.0
Middle	216	53.5
High	87	21.5
Oral health variable		
Cavitated dental caries		
Absent	260	64.0
Present	146	36.0
Outcome	Mean	SD
CPQ11-14	10.9	8.1

*Values lower than 406 are due to missing data. R$, Brazilian real (US$1.00 is equivalent to approximately R$5.1).

Table 2 summarizes the sample characteristics according to overall CPQ11-14 scores. Adolescents who rarely used social media had lower CPQ11-14 scores (mean 8.5; SD 6.0), indicating better OHRQoL. Additionally, those who had no one to trust online had higher scores (mean 11.5; SD 8.8). In terms of family income, individuals from higher-income families had lower scores (mean 10.1; SD 8.2). Adolescents who visited friends and family less than once a month or never had higher CPQ11-14 scores (mean 12.7; SD 9.9), as did those who did not feel supported during difficult times (mean 12.0; SD 10.0). Sense of coherence (SOC) was also associated with OHRQoL, with higher SOC scores corresponding to lower CPQ11-14 scores (mean 5.5; SD 4.2). Moreover, individuals without cavitated dental caries lesions had higher scores (mean 11.0; SD 8.4).

**Table 2:** Characteristics of the sample from Santa Maria, RS, according to overall CPQ11-14 scores, (n = 406).

Variables	CPQ11-14
	Mean (SD)
Demographic and socioeconomic variables	
Sex	
Boys	10.6 (7.9)
Girls	11.1 (8.3)
Age	
14–15	10.6 (8.1)
16–18	10.9 (7.8)
Household income quartiles in reais (R$)	
Quartile 1 Lowest	11.1 (8.1)
Quartile 2	11.8 (8.4)
Quartile 3	10.6 (7.6)
Quartile 4 Highest	10.1 (8.2)
Offline social capital	
Visit to friends/neighbors	
At least once a month	10.7 (7.9)
Less than once a month/never	12.7 (9.9)
Support during hard times	
Yes	10.7 (7.9)
Not	12.0 (10.0)
Online social capital	
Frequency of use of social media	
Every day or almost every day	10.9 (8.2)
Rarely	8.5 (6.0)
Never	11.8 (7.8)
Online social trust	
Yes	10.4 (7.7)
Not	11.5 (8.8)
Psychosocial variable	
Sense of coherence	
Low	16.5 (9.8)
Middle	10.3 (6.7)
High	5.5 (4.2)
Oral health variable	
Cavitated dental caries	
Absent	11.0 (8.4)
Present	10.6 (7.5)

R$: Brazilian Real (US$1.00 is equivalent to approximately R$5.1).

Table 3 shows unadjusted and adjusted regression analyses between online social capital, CPQ11-14 scores, and covariates. In the unadjusted model, adolescents who rarely used social media were less likely to report an impact on OHRQoL compared to frequent users (RR = 0.78; 95%CI: 0.63–0.95). Those without someone to trust online had a greater impact (RR = 1.10; 95%CI: 1.03–1.17). After adjustment, only online social trust remained significantly associated with OHRQoL (RR = 1.08; 95%CI: 1.01–1.15), suggesting that adolescents lacking online social trust had CPQ11-14 scores 8% higher, indicating worse OHRQoL. In addition, low offline social capital—characterized by not regularly visiting friends or neighbors and lacking support during difficult times—was also significantly associated with poorer OHRQoL. Additionally, individuals with moderate or high levels of SOC were less likely to experience negative impacts on OHRQoL compared to those with low SOC. Adjusted models presented lower goodness-of-fit values compared to crude models, suggesting an improved fit after the inclusion of covariates.

**Table 3:** Non-adjusted and adjusted analysis of the online social capital and overall CPQ11-14 scores, determined via Poisson regression analysis in a sample from Santa Maria (n = 406).

Variables	Non-adjusted		Adjusted	
	RR (95% CI)	p-value	RR (95% CI)^†^	p-value
Online social capital				
Frequency of use of social media				
Every day or almost every day	1 (reference)		1 (reference)	
Rarely	0.78 (0.63–0.95)	< 0.05	0.99 (0.78–1.27)	0.999
Never	1.08 (0.88–1.33)	0.421	1.12 (0.85–1.47)	0.402
Online social trust				
Yes	1 (reference)		1 (reference)	
Not	1.10 (1.03–1.17)	< 0.01	1.08 (1.01–1.15)	< 0.05
Covariables				
Sex				
Boys	1 (reference)		1 (reference)	
Girls	1.04 (0.98–1.10)	0.153	1.01 (0.95–1.08)	0.587
Age				
14–15	1 (reference)		–	-
16–18	1.02 (0.96–1.09)	0.374		
Household income quartiles (R$)				
Quartile 1 Lowest	1 (reference)		1 (reference)	
Quartile 2	1.06 (0.97–1.15)	0.147	1.18 (1.00–1.28)	0.050
Quartile 3	0.95 (0.88–1.04)	0.349	0.95 (0.87–1.04)	0.360
Quartile 4 Highest	0.91 (0.83–1.00)	0.050	0.98 (0.89–1.07)	0.680
Visit to friends/neighbors				
At least once a month	1 (reference)		1 (reference)	
Less than once a month/never	1.19 (1.08–1.31)	< 0.01	1.17 (1.08–1.28)	< 0.01
Support during hard times				
Yes	1 (reference)		1 (reference)	
Not	1.12 (1.00–1.24)	< 0.05	1.20 (1.07–1.35)	< 0.01
Sense of coherence				
Low	1 (reference)		1 (reference)	
Middle	0.62 (0.59–0.66)	< 0.01	0.62 (0.58–0.66)	< 0.01
High	0.33 (0.30–0.37)	< 0.01	0.32 (0.28–0.35)	< 0.01
Cavitated dental caries				
Absent	1 (reference)		1 (reference)	
Present	0.96 (0.90–1.02)	0.250	1.00 (0.94–1.07)	0.775
Goodness of fit	Crude		Adjusted	
Pearson χ^2^	2.444.453		1473.66	
Deviance	38.725.142		28.709.566	
AIC	3.874.514		2.896.957	

RR: rate ratio; CI: confidence interval; df: degrees of freedom; AIC: Akaike Information Criterion. Variables in bold indicate statistical significance.

## Discussion

This study assessed the relationship between online social capital and OHRQoL in adolescents. To the best of our knowledge, this is the first oral epidemiological study to explore this association. The findings partially confirm our conceptual hypothesis, showing that individuals with low online social trust exhibited poorer OHRQoL. However, the frequency of social media use was not associated with OHRQoL after adjustment for confounders.

Our results indicate that cognitive online social capital—specifically trust—was related to CPQ11-14 scores. Adolescents who lacked trust in their online communication partners reported worse OHRQoL. This is consistent with studies on offline social capital, which show positive associations between cognitive components like trust and better oral health outcomes, including self-rated oral health and quality of life.^32^ Although these studies were conducted in a traditional social capital context, the results can be extrapolated to the online context, as some authors suggest that classical definitions of trust can also be applied to trust in online relationships.^19^ This finding may be explained by several factors. Social trust is crucial for building social capital in adolescence.^32^ It acts as a protective factor by promoting positive relationships and developing social skills.^32^ Additionally, in the digital environment, young people feel more comfortable discussing sensitive topics, fostering greater openness and honesty than face-to-face interactions. This increased sense of security in the digital space allows adolescents to feel more confident in their social interactions and seek support when needed. Conversely, adolescents may report worse OHRQoL if they experience low social trust, as the lack of perceived social support and insecurity in social interactions can lead to feelings of isolation, low self-esteem, and emotional difficulties,^33^ negatively impacting their oral health.

In the context of oral health, trusted online contacts may also play a role in how adolescents cope with dental problems or aesthetic concerns. Adolescents may turn to these individuals to talk about pain, discomfort, or embarrassment related to their teeth, which could influence their willingness to seek treatment or adhere to oral hygiene behaviours.^9^ Additionally, peer advice or support received through digital channels might help adolescents deal with issues such as teasing or bullying about dental appearance, situations that are known to affect self-esteem and OHRQoL.^34^ Therefore, online trust may serve as emotional support and also as a buffer against negative social experiences that impact oral health perceptions.^32^ In addition, it is also important to consider the role of digital literacy in shaping online social capital and its potential effects on adolescent health.^35^ Adolescents with greater digital literacy may be more capable of engaging meaningfully with others online, navigating information critically, and avoiding harmful interactions.^35^ These skills can strengthen the positive aspects of online social capital, such as access to support and reliable information, potentially improving health outcomes.^15^ Future studies should explore how digital literacy interacts with online social trust and participation, and how this dynamic influences OHRQoL.

Despite its potential benefits, social capital is not universally protective and may also entail negative consequences. High levels of social capital may lead to adverse health outcomes, including behavioral contagion and interactions between social cohesion and individual characteristics. In the online context, this may manifest through cyberbullying, the spread of misinformation, or social comparisons, which can negatively impact adolescents’ well-being and oral health outcomes. For instance, online social networks may create environments where adolescents are exposed to unrealistic beauty standards or negative feedback, leading to lower self-esteem^34^ and, ultimately, poorer OHRQoL. Thus, while online social capital can be beneficial, it is crucial to consider potential negative effects, especially among adolescents.^36^

We also evaluated the structural component of online social capital—social media use frequency.^14^ Both excessive use and non-use were linked to worse OHRQoL. In this context, moderate social media use may act as a protective factor, highlighting the need for balance and quality in social media engagement. However, unlike social trust, the frequency of social media use did not remain associated with OHRQoL after adjustments. This may be due to socioeconomic differences, where adolescents from wealthier families have greater access to care, buffering any negative effects of high media use.^37^ Additionally, individuals with a greater SOC may be better able to cope with potential stressors associated with online interactions, such as cyberbullying, negative social comparison, or exposure to harmful content. As a result, more resilient individuals tend to feel less affected by oral health issues and consequently have better OHRQoL.^5^

In addition to online social capital, we also found that low offline social capital—marked by lack of social visits and insufficient support in difficult times—was associated with worse OHRQoL. Moreover, adolescents with moderate or high levels of SOC were less likely to report negative impacts on OHRQoL, supporting the protective role of psychosocial resources.^1,4,5,30^ On the other hand, cavitated dental caries and household income were not significantly associated with OHRQoL. This may be due to the distal nature of socioeconomic factors and social capital, which are conceptualized as structural determinants of health. Dental caries, in this context, may represent a mediating factor rather than a direct predictor—potentially influenced by social capital and, in turn, affecting OHRQoL. Thus, the integrative model adopted in this study allowed us to assess the association between online social capital and OHRQoL while adjusting covariates, reinforcing the independent role of social factors in shaping adolescents’ perceptions of oral health.

Moreover, adolescents with moderate or high levels of SOC reported better OHRQoL scores compared to those with low SOC, supporting the protective role of psychosocial resources^38^. In contrast, cavitated dental caries was not significantly associated with OHRQoL, which differs from some previous studies.^38,39^ This may be due to the relatively similar mean OHRQoL scores between children with and without caries, potentially limiting the detection of an effect. In addition, our robust multivariate model included several psychosocial variables, which are more distal determinants and may have attenuated the direct impact of caries on OHRQoL. Overall, SOC appears to buffer the potential negative effects of oral health problems on adolescents’ quality of life, reinforcing its importance in oral health promotion strategies.

This study has some limitations that should be acknowledged. Although based on data from a longitudinal study, causal inferences between online social capital and OHRQoL cannot be drawn. The items used to assess online social capital, while informed by previous research, were not cross-culturally adapted and covered only two dimensions—frequency of use and perceived trust—limiting the scope of the construct. However, questions used were based on prior studies that considered them proxy indicators of online social capital.^15,16^ Other relevant aspects, such as emotional support, access to information, and participation in online groups, were not explored and should be considered in future research. We also lacked data on specific social media platforms and time of use. Furthermore, data collection occurred during the national Multivaccination Day in Santa Maria, including only children attending the public campaign, which may have introduced selection bias. However, official data indicate that 90% of preschoolers in the city were vaccinated through the public health system that year, supporting the representativeness of our sample.

Although the differences in OHRQoL scores between categories of online social capital were modest—ranging from 1.5 to 3.5 points—they may still be considered clinically meaningful, as similar magnitudes have been reported in studies assessing the impact of dental caries and orthodontic treatment on adolescents’ quality of life.^28,40^ Considering the growing role of digital interactions in adolescents’ lives, these results highlight the importance of acknowledging social capital as a relevant determinant of oral health. Future research should prioritize longitudinal and mixed-methods designs, including qualitative approaches, to better capture the contextual and emotional dimensions of adolescents’ digital social interactions. Such studies may enhance our understanding of these associations and support the development of targeted interventions within digital health strategies.

## Conclusion

Our findings suggest that online social trust, offline social capital, and sense of coherence play important roles in adolescents’ OHRQoL, whereas the frequency of social media use showed no significant association. These results highlight the need to integrate digital literacy, social trust-building, and psychosocial support into clinical programs (e.g., preventive and educational oral health initiatives) and public health policies (e.g., school-based health promotion, digital literacy campaigns, and adolescent psychosocial support programs) aimed at promoting adolescents’ well-being.

## Data Availability

The datasets generated and/or analyzed during the current study are available from the corresponding author upon reasonable request.
